# Supplementary material for: Exploring use of coercion in the Norwegian ambulance service – a qualitative study
Source: Scand J Trauma Resusc Emerg Med. 2023 Sep 4;31:44. doi: 10.1186/s13049-023-01104-x (PMC10478396; doi:10.1186/s13049-023-01104-x)
Supplement: Supplementary file 1 — Supplementary Material 1 [file 13049_2023_1104_MOESM1_ESM.docx]

# Exploring use of coercion in the Norwegian ambulance service

# – a qualitative study

Nina Oeye Thorvaldsen^1,2^ Tonje Lossius Husum^1,3^  Stephen J.M. Sollid^2,4^

Address: Faculty of Health Sciences, Oslo Metropolitan University^1^ , Faculty of Health Sciences, University of Stavanger^2^, Centre for Medical Ethics, University of Oslo ^3^, Prehospital Division, Oslo University Hospital^4^

Email: Nina Oeye Thorvaldsen*- [nithor@oslomet.no](mailto:nithor@oslomet.no); Tonje Lossius Husum- –tonjelos@oslomet.no

Stephen J.M. Sollid – uxsosb@ous-hf.no

*Corresponding author

## Abstract

### Background

Healthcare laws allow for exceptions from the consent requirement when patients are not competent to consent or pose a danger to themselves or others. In these cases, the use of coercion may be an alternative to voluntary health care. Ambulance personnel are regularly confronted with patients who need healthcare but refuse it and/or refuse to cooperate. This study aimed to explore ambulance personnel`s experience with use of coercion and factors influencing the use of coercion in the ambulance service in Norway.

### Method

We conducted two focus group interviews with a total of eight informants, all ambulance personnel from a large Norwegian ambulance service. Digital recordings of the interviews were transcribed verbatim and analysed using systematic text condensation.

### Results

The informants` stories revealed several methods of coercion used by the ambulance personnel; physical coercion, pragmatic coercion, pharmacological coercion and coercion used to ensure the patient is secured during transportation. The main reasons for using coercion were preventing patients from harming themselves or others and to ensure that patients unable to consent receive healthcare considered necessary. Systemic factors as difficulty of applying the law to real-life situations, and organizational factors as fear of breaching guidelines, experienced lack of support from the management, fear of charges of misconduct, and lack of training in assessing patients´ competence to consent seem to influence ambulance personnels use of coercion.

Conclusion

Ethical grey areas in clinical practice emphasize the need for clinical discretion. Despite the fact that regulatory provisions allow for exceptions from the requirement to obtain consent, transferring these regulations to real life prehospital settings can be difficult. Consequently, the decisions made by ambulance personnel in clinical situations are highly influenced by organizational ethos and guidelines. The informants describe the coercive interventions they have employed to manage patients who are deemed to require healthcare but refuse it and/or refuse to cooperate.

### Keywords

Ambulance, coercion, competence, focus groups, healthcare, informed consent, Norway, patients, prehospital

## Introduction

Voluntary and informed consent is a fundamental principle of healthcare provision. The use of coercion represents a significant intrusion in an individual’s life, violating both legal and ethical principles concerning the right to freedom and self-determination (1, 2). Modern health legislation generally defines coercion as the interaction between health personnel and patients that necessitates safeguarding by the rule of law from the patient's perspective (3). Although Norwegian health legislation does not provide a single, cohesive definition of coercion, it is recommended to define it as "overcoming resistance." (3). As a general principle, coercion, specifically physical coercion, remains a task reserved for law enforcement agencies. However, healthcare laws in Norway provide exceptions to consent requirements and allow for the use of coercion under certain circumstances, such as when patients are incompetent, unable to provide consent, or pose a danger to themselves or others (4-7). Ambulance services, being at the forefront of prehospital care, often encounter situations where these exceptions may apply. However, Norwegian regulations have not been specifically designed, developed, or discussed in the context of the prehospital setting (8, 9). The Health Personnel Act section 7 instructs health care personnel to provide care they are capable of, if it is considered of vital importance for the patient (10). This applies even if the patient is incapable of providing consent or objects to the treatment. In practice, section 7 is often perceived as a legal basis for coercion, but it has faced criticism from a legal standpoint (11). The Patient Rights Act section 4 A allows for coercion if a patient requires somatic healthcare, is incompetent, and refuses healthcare (12). Certain conditions must be met before employing coercion under section 4 A, such as attempting confidence-building measures, assuming that failure to provide healthcare will result in significant harm, considering healthcare necessary, and ensuring the measures needed are proportionate to the health care required (12). The Health and Care Act regulates coercion against persons that are intellectually disabled or abuse substances (7). The Mental Health Care Act section 3-2 and 3-3 allow for physicians to commit patients to forced mental healthcare if the patient is not competent or poses a threat to their own life or the health and/or lives of others (6, 13). The municipal chief medical officer or his or her deputy can on their own initiative or at the request of another public authority or next-of-kin, decide that a medical exam is needed and that the exam can be done by force if necessary (14). The use of mechanical coercive means or single doses of short-acting drugs for the purpose of sedating or anesthetizing the patient, is only regulated inside mental health hospitals (15). The Norwegian Penal Code section 17 and 18 provides for actions deemed necessary to save lives or protect others from harm (16).

In some countries, guidelines exist for the use of short-acting drugs in prehospital settings when patients physically resist healthcare. For instance, the Royal College of Emergency Medicine in the United Kingdom (UK) has developed guidelines for the management of excited delirium and/or acute behavioural disturbance which are characterized by “the sudden onset of aggressive and violent behavior and autonomic dysfunction” (17). Excited delirium and acute behavioural disturbance are considered medical emergencies, as affected individuals may experience sudden cardiovascular collapse and/or cardiac arrest. The guidelines emphasize that physical restraints could exacerbate autonomic dysfunction and should be kept to a minimum (17). The National Organization of EMS Physicians (NAEMSP) in the United States of America (USA) recommends that prehospital services formulate protocols consistent with legislation and local guidelines, including type of and use of restraint; verbal, physical, and chemical (18). Pharmacological coercion or chemical restraint in the prehospital setting has been discussed in studies from the USA and Spain (19-24) with the focus mainly on the most suitable sedative for prehospital use. To the best of the authors` knowledge, no guidelines, recommendations, or studies on pharmacological coercion or chemical restraint have been conducted within prehospital services in Norway.

Norway has 5.3 million inhabitants unevenly distributed on 323 800 km^2^. The healthcare system is publicly funded, based on the principle of universal access, and financed through taxes as well as employer and employee payroll contributions. Although emergency care is provided free of charge, other services require a small co-payment with caps on out-of-pocket expenses. All 18 ambulance services in Norway are organizationally part of a local health trust and generally function as independent organizations, with a varying degree of inter-service cooperation e.g. regarding guidelines (25). Ambulance personnel in Norway possess delegated rights to administer certain medications. These delegated rights vary across ambulance services and depend on the level of training, competence, and experience. Delegations are authorized by the operation manager and an assigned medical advisor. Additionally, a physician can prescribe and delegate the administration of additional medication to ambulance workers under the individual physician`s responsibility. All admissions to mental health hospitals require a referral from a physician (6), whereas critically ill and injured patients can be directly admitted by ambulance personnel (26). If the patient does not need to be admitted, they can be referred or transported to a consultation by a regular general practitioner (RGP) or a physician from out-of-hours emergency primary health care. If the patient refuses healthcare (including transportation to a physician), guidelines in some ambulance services demand that ambulance personnel consult a physician by phone. However, ambulance personnel have an independent responsibility to conduct their work in accordance with professional responsibility and diligent care requirements (4).

The use of coercion in Norwegian ambulance services is poorly documented, primarily due to the existence of separate databases for each service and the lack of uniform data registration methods (25). Available data indicates that coercion is employed in 4.5-10.3% of ambulance missions involving patients with mental health problems (27-32). Despite its prevalence and application, no prior research on this topic had been conducted when this study was carried out in 2019. This study aimed to explore ambulance personnel`s experience with use of coercion and factors influencing the use of coercion in the ambulance service in Norway.

Method

A qualitative research design utilizing systematic text condensation (STC), a method developed by Malterud, was selected due to its exploratory nature and suitability for novice researchers (33, 34). STC is grounded in Giorgi’s psychological phenomenological method yet adopts a more pragmatic and descriptive approach (34, 35). Data were collected through two focus group interviews; one pilot interview and one primary interview. Focus group interviews were chosen for several reasons: 1) Malterud (2012) recommends focus group interviews in research with an explorative design where the aim is to describe or understand 2) conversational exchange gives the opportunity to collect not only meanings and experiences but also contextual details that shape meaning construction (36); and 3) group dynamics provide the advantage of one story triggering associations in other informants, thereby generating more stories and richer data (37).

### Recruiting and interviews

Ambulance personnel (Ambulance Service Technicians and paramedics) employed by the Division of Prehospital Services at Oslo University Hospital (OUS) constituted the population. The division employs approximately 900 individuals across 18 ambulance stations. The pilot interview involved three strategically selected ambulance personnel with varying levels of experience and education. After conducting the primary interview, the authors decided to include the data from the pilot interview in the final analysis, as it provided valuable insight.

The primary informants in this study were predominantly recruited through the managers of the ambulance stations. An email was sent to each station manager, containing an invitation to participate in the study, along with a request to inform their employees and post the invitation on a noticeboard. The invitation provided details about the study and contact information for the first author. In response, Six employees volunteered to take part in the primary interview. One employee withdrew from participation due to a combination of a lengthy journey and illness.

A total of eight informants from three different ambulance stations, five men and three women, with between 1.5- and 28-years’ experience of the ambulance service, took part in the focus groups. One informant had a bachelor`s degree in paramedicine, two were Ambulance Service Technicians with additional paramedic-level training and a bachelor`s degree in nursing, three were Ambulance Service Technicians with additional paramedic-level training, one was an Ambulance Service Technician, and one was an Ambulance Service Technician with a bachelor’s degree in nursing. The informants are not described further for reasons of anonymity.

The interviews took place in autumn 2019 in a meeting room in a public building with the first (NOT) and second (TLH) authors present. Using a semi-structured interview guide based on the study's aim (appendix 1), the first author (NOT) facilitated the interviews. Meanwhile, the second author (TLH) took notes and sought clarifications as needed at the end of each interview. The pilot interview lasted 90 minutes, and the primary interview lasted 100 minutes. Both interviews were recorded and later transcribed verbatim by NOT.

### Ethics

The Regional Committees for Medical and Health Research Ethics (reference 2019/927/REK sør-øst) deemed that the study was outside the scope of the Health Research Act. The Norwegian Centre for Research Data (reference 718167), the Data Protection Officer at OUS, and the management of the ambulance service at OUS granted their approval of the study.

All informants provided signed consent declarations and were encouraged to treat the information disclosed in the interview as confidential. The interviews were recorded using two separate devices not connected to the internet. The audio files were encrypted using the program VeraCrypt, 265 bits AES encrypting, before being transferred, and the original audio files on the recording devices were subsequently deleted. The interviews were anonymized during the transcription process. To further safeguard the anonymity of both the informant and third parties, the stories shared by the informants were altered, and two of the stories from informants 3 and 4 under the heading "pragmatic coercion" in the results section were shortened. To further ensure anonymity, all informants will hereafter be referred to as ‘him’ and information about the informants will be kept to a minimum. During transcription, one informant was contacted twice to confirm our interpretation of his statement. After transcription the audio files were deleted.

### Analysis

STC`s four steps were closely followed.

**Step 1: From a bird’s eye perspective to preliminary themes**

Both interview transcripts were read independently by the first author, NOT, and a colleague, JJ. The purpose of involving JJ in this process was to ‘create a wider analytic space’ (33). The two identified themes and key words that seemed important in an inductive approach. The keywords and themes from the two interviews were compared and merged to form preliminary themes. The remaining analysis was performed solely by NOT.

**Step 2: Identifying and sorting meaning units – from preliminary themes to codes**

The preliminary themes were considered against the study´s aim and the questions from the interview guide. Consequently, the preliminary themes were revised into codes, which represented more overall topics. The transcripts were read through, and meaning units, i.e., text fragments containing information about the codes, were assigned to the corresponding code using ´copy and paste´ in word. Table 1 provides an overview of the first two steps of the analysis.

Table 1: Overview of the first two steps of the analysis

| **Preliminary themes** | **Codes** | **Examples of topics sorted under the codes** | **Subgroups** |
| --- | --- | --- | --- |
| Patients who refuse healthcare  Assessment of patients competence to consent | Experience with using coercion (stories) | - Removing razor blades or knives - The physician does not come to the patient, the patient is taken by force to be assessed by a physician - To secure patients with that physically act out during transportation - Sedate patients without consent because of necessity or safety - Principle of necessity suspecting the patient is intoxicated, has hypoglycemia, an infection, a psychiatric condition or other | - Principle of necessity - Legal duty to care |
|  | Situations where coercion was used | - Involuntary admission - Suicide risk - Patients who can`t fend for themselves - The physician does not come to the patient, the patients need to be assessed by a physician - Securing the patient during transport - The patient does not realize the seriousness in the situation |  |
|  | Forms of coercion | - The patient is not given a choice - Leading the patient by the arm - Aid the police in restraining and securing the patient - Hold an arm, sit on the patient - Wrap a blanket around the patients’ arms with the security belt tightly fastened around the patient | - Pragmatic coercion - Securing the patient during transport - Pharmacological coercion - Physical coercion |
| Guidelines versus the rules and regulations | Factors affecting the use of coercion | - Insecurity about how to apply the legislation to real life situations - Interpretation of the guidelines-all patients should be assessed by a physician - When a medical physician does not respond to the AP* call, they feel forced to bring the patient to the Emergency Medical Scenter - A perceived lack of support from the management - Increased fear of making mistakes | - Systemic factors   - Legislation - Organizational factors   - Guidelines   - Culture of fear   - Insecurity |
| Concern for consequences |  |  |  |

Both interview transcripts were read while noting themes and key words. Keywords and themes from the two interviews were compared and merged to form preliminary themes. The preliminary themes were considered against the aim and the questions from the interview guide and revised into codes. Meaning units, i.e., text fragments containing information about the research questions and chosen codes, were assigned to the codes using ‘copy and paste’ in Word, here with examples of topics sorted under each code. Each code was divided into 2–4 subgroups. For an overview of how themes, codes, and subgroups are connected, see figure 1. *AP- ambulance personnel

**Step 3: Condensation – from code to meaning**

Each code was divided into 2–4 subgroups to differentiate topics within each code. The meaning units were sorted accordingly. Codes and subgroups were merged and meaning units relocated during an ongoing dynamic process at this stage. The meaning units of each subgroup were then reduced and abstracted into a condensate – an artificial quotation that maintained the terminology used by the informants. The respondents´ stories sorted under the code “experience with using coercion” proved difficult to reduce into a condensate, so the meaning units were relocated to other codes and the condensational process was redone.

**Step 4: Synthesising – from condensation to description**

An analytic text was synthesised from the condensate for each subgroup, forming the basis for the result sections. Authentic quotes were selected to represent and complement the analytical text.

# Results

The code “factors affecting the use of coercion” is employed at the first headline in the result section. The code “forms of coercion” serves as the second heading with its subgroups functioning as subheadings. The codes “experience using coercion” and “situations coercion used” do not have their own headings; however, the respondents´ descriptions sorted under these codes are distributed as explanations and examples throughout the result section.

Figure 1 gives an overview of the result as an overview of how the connection between the results can be viewed.

Figure 1.

Title: Factors that influence how ambulance personnel handle patients who refuse healthcare

Legend: The circles indicate that organizational factors (factors within the organization), systemic factors (conditions outside the organization) (38), and real-life situational factors affect how ambulance personnel evaluate and respond to situations in which patients refuse healthcare, leading in some cases to the use of coercion to complete the assignment.

Factors affecting the use of coercion

The use of coercion in prehospital real-life situations was found to be highly influenced by systemic factors, elements outside the organization as the legislation and organizational variables, factors within the organization as guidelines, insecurity, and a culture of fear. The majority of the informants seemed to adopt a practical approach towards the use of coercion, primarily regarding it as the application of physical force when deemed necessary to fulfill their duty to care or to protect the patient or themselves from harm. Informants reported using physical coercion to disarm patients or remove objects used for self-harm, such as razor blades or knives. There were instances when patients became extremely agitated during transportation; in one case, the ambulance crew had to evacuate and leave the patient in the ambulance due to conscerns for their own safety. In other situations, the ambulance personnel opted to use physical coercion to restrain the patient until police assistance arrived. One example involved a patient who lost control of himself because of presumed intoxication:

Informant 4: On the two occasions I have fought with someone at work, it was due to intoxication.

Interviewer: It´s become physical?

Informant 4: Yes. I had to hold them down, or they`d rip the ambulance apart. But it wasn`t really psychiatry, but it became psychiatry, but it was intoxication…triggered by intoxication.

The informants were conscious of their legal duty to care as healthcare professionals.

…and then there is our duty to care as healthcare professionals. We are summoned, we are healthcare professionals and therefore have a duty of care. (Informant 2)

The informants reasoned that they sometimes had to employ coercion based on their professional judgment, in the patient`s best interest, while adhering to relevant guidelines and legal frameworks. They recognized that some patients lacked the awareness or capacity to understand their need for medical attention or the risks associated with non-cooperation, which could be attributed to a variety of factors such as hypoglycemia, dementia, a brain hemorrhage, severe infection, intoxication. In these cases, the informant’s narratives illustrated that physical coercion was deemed necessary to ensure the provision of adequate treatment.

During the interviews, it became evident that the informants felt strongly bound by the service’s guidelines in their clinical practice. Simultaneously, they admitted to having limited awareness of the specific legislation governing their practice.

Interviewer: …when you make that judgement…do you consider that we have guidelines …and then we have the law, do you have any…what does your assessment look like…?

Informant 4: No, I don’t think about the law really, not that there are any provisions that I should…It is more like you should do as [name of person in management] says, that we should never leave a patient at home. That’s enough for me.

I feel like we are in a gray zone in a way, we are not certain what the law says, what the guidelines…where should we place ourselves? I have not received proper training…under certain circumstances, this or that should weigh more…and we very often state that the guidelines state that we should not leave anyone at home… (Informant 1)

Informants frequently reported that adherence to their interpretation of local guidelines was a key factor influencing the use of coercion in their clinical practice. They expressed an increasing concern about breaching guidelines and facing charges of negligence, particularly in light of high-profile cases involving ambulance personnel being charged with misconduct and the subsequent negative media attention that followed (34, 35). The informants reported hearing accounts of inadequate management support following charges of misconduct, causing them to feel apprehensive about leaving a patient at home/at the scene despite the patient`s explicit refusal of further medical care. This apprehension was underpinned by the informants' uncertainty regarding management`s backing in cases where they might be charged with negligence or misconduct.

… should something happen, the management won`t support us, they will probably back off and say we didn`t follow the guidelines, so… (Informant 2)

Although the informants stated that they were more reluctant to use coercion on a patient they deemed competent to consent, they acknowledged that their training in assessing a person’s capacity to consent was limited. They expressed uncertainty regarding the interpretation and application of the legislation in everyday decision-making. The informants felt they needed more knowledge about the legislation to effectively use it in real-life situations. They had numerous legal questions they wished to clarify and discuss, but encountered difficulties in obtaining clear answers from management when they approached them with inquiries and concerns.

Informant 7: I feel like we are put in a role … we are obliged to solve a task that cannot be … solved because they [the management] are not willing to address it. They [the management] just look the other way and count on us to figure out something smart. And if something goes wrong, presumably you must account for it.

Interviewer: … so then they would rather lose you?

Informant 7: yes

The informants’ experiences indicated that neither education, training, the system, nor the legislation provide them with adequate guidance or support. One informant mentioned that, while their education focuses on caring for the acutely and critically ill or injured, the majority of patient cases encountered were related to general practice medicine, geriatrics, and mental health. This discrepancy was described as a source of frustration for the informant.

## Forms of coercion described

Although coercion primarily was regarded as physical force, the informants described different methods of coercion through their stories. These methods and uses of coercion described could be synthesised into four categories: physical coercion, pragmatic coercion, securing the patient during transport, and pharmacological coercion. Descriptions of physical coercion will not be further elaborated since covered in the section above. The other categories are described further in the sections below.

### Pragmatic coercion

The term “pragmatic coercion”, coined by the first author, refers to methods employed to circumvent the use of physical coercion. These methods include leading and guiding the patient, utilizing authority, employing white lies, and encuraging voluntary compliance through persuation. The objective is to presuade patients to accept transportation to one of three possible destinations; their RGP, out-of-hours emergency primary health care, or an emergency ward to be assessed by a physician. The ambulance service’s guideline (appendix 1) was cited as the rationale for employing these persuasion techniques, along with the management`s stated policy that no patients should be left at home.

If there had been a guideline that said that we could assess whether patients could stay at home, then things would have been different, but we don`t have that possibility because our manager says that we must not do it [leave patients at home]. (Informant 7)

Leading or guiding a patient to the stretcher or the ambulance was not considered coercion by the informants. They explained that their threshold for leading or guiding patients was higher when the patient was deemed competent compared to an incompetent patient.

If you have a patient you consider to be incompetent, who can`t take care of themselves, then we have to get them help. And if they resist for some reason, we have to take it one step further… (Informant 2)

Ambulance personnel may exhibit creativity when encountering patients who refuse healthcare and are deemed incompetent to provide consent in order to avert the need for physical coercion. Two examples from the interviews, shortened and altered to safeguard anonymity, illustrate this:

A demented patient in a nursing home refused to be transported to hospital after suffering a possible head injury. Eventually, a physician was summoned, who arrived at the nursing home with a taxi driver who was a huge, rugged man with a beard. After observing the situation for a while, the taxi driver offered to talk to the patient. He stood up and said, ´Listen here, you need to go with these gentlemen here.’ The patient immediately changed attitude and responded, ‘yes, yes’ and willingly went with the ambulance personnel to the hospital. (Informant 3)

A confused elderly person was found in a car park on a cold winter day. We were dispatched, but upon arrival, he refused to enter the ambulance. We called a relative and, based on information given by the relative, managed to convince the elderly person, by lying, that we would drive him to a Sunday dinner at the relative’s home. There was some commotion when he was taken to the nursing facility where he resided. However, if we had not done it, he would have fallen on the ice, sustained a femur neck fracture, and frozen to death or caught pneumonia. (Informant 6)

In the study, a ’white lie’ was identified as a method of pragmatic coercion aimed at overcoming resistance without resorting to physical force. When mental illness was suspected or the patient had expressed suicidal intentions, the informants perceived it as particularly important that the patient be assessed by a physician. However, they preferred to avoid using physical force or seeking assistance from the police and instead would give the patient an ultimatum: either come with the ambulance personnel ‘willingly’ or face interventions from the police. This approach, involving the use of threats or verbal coercion, was referred to as ‘voluntary coercion’ by one of the informants. The goal was to prevent the use of physical force, which was considered a last resort.

Handling patients who physically resisted and did not respond to verbal instructions, such as some patients with dementia, proved challenging. In one instance, an informant described how they avoided a punch and restrained the patient’s arm by holding it along the patient’s side. One informant suggested wrapping a blanket around the patient’s arms (possibly with a safety harness strapped over the blanket) as an alternative method of restraint for unruly or intoxicated patients. This same method was employed as a hygienic measure in a case where a patient was ‘fiddling’ with herself:

Informant 5: Yes, it sure was a long time since she had a shower. And she smelled, and she was going to… those hands, which had been deep down her trousers and… other places as well, and then she reached out to touch our faces. Oh my…

Informant 7: (inaudible)

Informant 5: it was a short process. We just ‘smick smack’ (miming wrapping a blanket around the patient) and down the stairs she went [she was carried down the stairs with a blanket wrapped around her arms].

### Securing the patient during transportation

Several of the informants reported assisting the police in applying physical force to patients who physically resisted healthcare during ambulance transports on multiple occasions. They mentioned using aids such as bandages, Velcro straps (backboard straps), or a vacuum mattress with ancillary straps.

Interviewer: Have you ever used spider straps [Velcro straps] to secure an agitated patient to the stretcher?

Informant 4: Mm-hmm.

Interviewer: But why? Was it because the safety harness wasn't enough?

Informant 4: Mm-hmm.

Interviewer: So, you're not able to keep an agitated patient restrained?

Informant 4: No, they loosen the safety harness. If you bind their arms down like this [demonstrates straight arm positioning] and secure everything over them, then they lie there. That way, they can't get anywhere.

Interviewer: So, why do you do it? Is it for traffic safety? To prevent self-harm? To ensure our safety or what is it that…?

Informant 4: All

Interviewer: All... All of the above?

Informant 4: Don't want them performing acrobatics in the vehicle. Can't have them loosening the safety harness while we're driving. When they lie there… in case of a collision, they remain securely tied down, unable to cause disruptions in the vehicle or harm themselves or others.

Furthermore, if a patient managed to free themselves from the safety harness, the accompanying police officers would often unbuckle their safety belts to physically hold the patient down. However, if the patient was adequately secured, the police could remain seated with their safety belts fastened. Some informants reported using similar methods to secure patients without the presence of police officers.

### Pharmacological coercion

One informant discussed an incident where diazepam was given to a patient without their consent because the patient posed a safety risk to themselves and others during transportation. Another story about a critically ill patient illustrates the use of pharmacological coercion:

This patient was septic and, due to low blood pressure, very confused. He refused to accompany us, but he was…we all agreed that he had to be taken to hospital, but he was large and located on the second floor, so there was no way. We enlisted the help of the fire brigade. The air ambulance was unavailable because they were occupied with other critical patients, so we gave him a good dose of diazepam to at least enable us to carry him down the stairs. He nearly died from meningitis. (Informant 6)

The informants discussed whether sedation might be a preferable option for certain patients rather than subjecting them to extreme physical coercion. For instance, they discussed a case involving a patient with a history of sexual abuse, suggesting that sedation could have been a more suitable approach instead of physically restraining the patient with the assistance of police officers during ambulance transportation.

## Discussion

The use of coercion in the ambulance service manifests in various forms, including pragmatic coercion, physical coercion, pharmacological coercion, and coercion employed to secure patients during transportation. Systemic, organizational, and real-life situational factors influence how ambulance personnel manage patients who refuse healthcare. Three aspects seem to particularly promt the use of coercion: preventing patients from harming themselves or others, ensuring that incompetent patients receive necessary healthcare and avoiding breaches of guidelines and charges of misconduct. The latter theme was consistently noted during the interviews. Patients who refuse healthcare underlie all examples and situations where coercion was utilized by the informants.

Patients who refuse health care present a dilemma for ambulance personnel. The service guideline “Patients who are not transported to hospital/physician” (appendix 1) is interpreted as “all patients should be assessed by a physician” and the ambulance personnel feel strongly bound by the guidelines. Consequently, they are hesitant to leave a patient if a physician cannot be summoned to the scene. However, regulations mandate that healthcare should be based on voluntary and informed consent (5) which means that if the ambulance personnel do not respect the patient´ choices, possibly due to a lack of awareness and knowledge of the legislation, they risk infringing on patients’ rights. Furthermore, assessing and documenting patients´ consent is considered vital to avoid legal repercussions (39-43). However, determining a person`s capacity to consent can be challenging, and ambulance personnel report limited training in assessing a patient’s capacity to consent, especially in cases involving mentall illness (40-43). In situations where the patient refuses healthcare and is assessed as incompetent to consent ambulance personnel seem to be prone to utilizing various forms of pragmatic coercion. Pragmatic coercion involves methods used to compel patients to receive healthcare without resorting to physical force. In mental health care context, ‘pragmatic coercion’ resembles the term’ informal coercion’ which encompasses behaviors such as persuasion, interpersonal leverage, inducements, threats, deception, employing a disciplinary style, and referring to rules and routines (44). The Norwegian Patient Rights` Act, Section 4A-4, permits the provision of healthcare by force or other means if the patient is incompetent, resists treatment, and the treatment is regarded as necessary to preserve life and prevent harm (5). In this context, the use of authority and occasional ‘white lies’ by ambulance personnel can be considered measures that align with the aforementioned legal provision.

Several of the informants’ stories reference physical coercion used as a safety measure, utilized to provide vital healthcare, protect the patient and ambulance personnel from harm, and ensure patient safety during transportation. This is carried out both with and without the presence ot the police. An alternative to physical coercion is medical sedation, which has been termed `pharmalogical coercion` in this study. Although there is no formal protocol for sedation in the pre-hospital setting in Norway, the practice of using medical sedation as an alternative to extensive physical coercion is accepted in some countries. For instance, recommendations from NAEMSP in the USA and guidelines from the Royal College of Emergency Medicine advocate for medical sedation to facilitate potentially lifesaving treatment and prevent physical injury in patients exhibiting aggressive and violent behaviour. This approach is also considered a safety measure for the ambulance personnel, who are obliged to aid, but generally are not trained in the use of physical force (17, 18). The use of sedatives on patients exhibiting physically aggressive behavior in prehospital care in Norway has not been widely debated. Nonetheless, the informants reported that transporting such patients safely presented practical and ethical challenges. In some instances, they assisted the police in exercising force on combative patients during ambulance transports, using equipment from the ambulance to secure the patient. Their objective was to ensure both the patient and the police were adequately secured during transport, reducing the risk of severe injury in the event of emergency braking or a road accident. This practice is documented in a recent study (8). Given that proper securing of drivers and passengers can reduce the risk of death or severe injury by 44-60% in the event of an accident (45, 46), this course of action seems reasonable. However, the legal basis for using coercion to protect passengers from potential injury in the case of an accident remains unclear. As a result, there are no guidelines or standardised methods to inform those involved in executing these assignments in a safe and dignified manner for the patient, ambulance personnel, and police. This may imply that those involved must improvise, weighing safety considerations against their uncertainty about the legal basis every time such a situation arises. The use of physical coercion needed to secure these patients contrasts with the Royal College of Emergency Medicine´s recommendations for treating acute behavioural disturbance or excited delirium, which suggests that physical restraint can pose a threat to the patients (17). During a discussion among the informants regarding the patient´s best interest, a girl with a history of sexual abuse was used as an example. They pondered whether pharmacological coercion would be a better alternative for some patients than having police officers physically restraining them. Studies in the mental healthcare field have shown that patients have both positive and negative experiences with the use of short-acting sedatives, although these sedatives appear preferable in crisis situations (47). We reason that if pharmacological coercion is given outside a mental hospital in Norway, its legality must be based section 7 of The Health Personnel Act (10). If used to save life or health or to protect others from harm, the Norwegian Penal Code, Chapter 3, Section 17 or 18 can be applicable (16).

The informants’ stories indicate that police assistance is primarily requested when the presumed cause of the patient´s behavior is related to a mental health condition and/or substance abuse. Consecuently, the informants appeared more inclined to use coercion on patients with presumed somatic illnesses without police assistance. A possible explanation for this could be the reported inadequacy in assessing patients with mental health problems, as well as the perception that patients with mental health and/or substance abuse issues are considered more unpredictable in their behavior (40, 41). This likely reinforces the sense of insecurity surrounding these patients and lowers the threshold for requesting police assistance. Given that the informants´ education primarily focuses on caring for acutely and critically ill or injured somatic patients, it is reasonable to assume that they find such patients easier to assess. Although the informants generally find it difficult to apply the regulatory provisions that allow for exceptions from the consent requirement to real-life situations, they appear to be aware of their legal duty to provide care when they deem healthcare to be of vital importance to the patient (10). Inadequacy in assessing patients with mental health issues likely make it more difficult to determining when these patients require urgent care. Even mental health specialists appear to have difficulty determening when a mental health conditions poses a life threatening risk (48, 49).

Studies related to prehospital transport of patients with mental health issues in Norway have primarily focused on enhancing the healthcare personnel´s competence (29, 50, 51). This may indicate that the education provided does not sufficiently equip ambulance personnel to handle situations involving patients requiring mental healthcare. Developing the competence and skills of health care personnel has been shown to result in a reduction in the use of coercion in mental hospitals (52), and it has been emphasized as a key factor in the commitment to further reduce coercion in compulsory mental healthcare (53). The absence of adequate competence, training, and feedback on performed clinical practice may lead ambulance personnel to base their professional practice on lessons learned from supervisors, co-workers, and experiential learning. Measures, such as securing patients exhibiting physical aggression with bandages and Velcro straps, can become established practices if not corrected and if a lack of alternatives persists.

In situations involving patients with suspected mental health illnesses, a variant of pragmatic coercion was described. The term ‘voluntary coercion’ was used to characterize efforts and attempts to persuade a patient to comply without using physical force. The informants did not necessarily perceive it as a threat or coercion, but rather as a pragmatic solution to the situation. Typically, this ‘method’ is used to ensure compliance with the service’s guidelines and that the patient is assessed by a physician. Under the Mental Health Act 2007 in Australia, ambulance officers are permitted to detain patients who appear to be mentally ill or mentally disturbed if it is considered beneficial for the patients` welfare (42). Only 27% of the patients detained by Australian ambulance officers were admitted involuntarily, according to a study examining the involuntary admission rate (42). The Australian ambulance officers characterized patient detention as a crucial measure for ensuring the patients’ further assessment and safety. Nearly half of the detained patients were identified as suicidal or as having self-harmed. A UK study examining paramedics’ perspectives on the care they provided to patients who self-harmed reveals parallels (43). Similar to our findings, the study found that paramedics were willing to deny competent patients their right to refuse healthcare for paternalistic reasons and ‘nobody dies on my watch’ mindset. This attitude may be based on a number of factors, including concern for the patient (42, 43), guidelines that are inconsistent with the law (43, 54), legislation that is difficult to apply in real-life situations (40, 42, 55), fear of negligence (43, 54), lack of knowledge (40, 43, 56) and a perception of lack of support from the management or the organization (43, 56). A sense of conflict and isolation when making decisions regarding the use of coercion was a recurrent theme in the data, and has also been described by others in similar settings (43). We are concerned that health care personnel`s fear of negligence may be exacerbated if they do not percieve the support of their organization`s management, particularly when facing a conflict between guidelines and legislation. Contradictions between guidelines and legislation are something that are known and problematic (43, 54).

## Limitations

This study was limited to the ambulance service at one health trust`, and the findings may have limited transferability to ambulance services in general. However, we note that several of the identified challenges have been addressed by other authors in different countries, indicating that some of the challenges related to coercion in the ambulance service may be universal in many respects. Particularly, the percieved lack of competence regarding patients with mental health illnesses seems to be a pervasive issue.

One aspect not addressed in our study was the difference between urban and rural areas, and the impact of this factor on the ambulance personnel`s decision making remains unknown. In qualitative studies, there is a general recognition that the researcher’s preconceptions, knowledge, and experience will affect the research process (30). The first author’s experience as a paramedic formed the basis for the study, and it is plausible that the role as researcher and colleague provided access to data that might be difficult for others to obtain. By being aware of her preconceptions, collaborations with supervisors, and maintaining methodological rigour, the first author has striven to do justice to the informants’ stories and opinions.

## Conclusion

This study examines the use of coercion in various situations in a Norwegian ambulance service. The informants discussed instances involving patients who refused health care. The ambulance personnel`s use of coercion was influenced by systemic and organizational factors, as well as factors in the real-life prehospital situation. Two main factors found were the difficulty in transferring statutory consent requirements to real-life prehospital situations (systemic factor) and the perception that guidelines demand that all patients be assessed by a physician (organizational factor). We identified four modes of coercion: physical coercion, pragmatic coercion, pharmacological coercion and securing during transport. In general, coercion is employed in the patient`s best interest and/or as a security measure to protect both the patient and the ambulance personnel from harm.

The identified shortcomings in prehospital personnel`s knowledge and awareness of the appropriate use of coercion and legislative restraints could potentially undermine patients' legal rights. We suggest incorporating these topics more effectively into both basic training and continuous professional development curricula. The legal basis for necessary coercion to secure patients who physically resist healthcare during ambulance transports, despite its vital importance for patient safety, lacks clarity and demands immediate attention. Additionally, it is crucial to clarify the practical aspects of handling patients who resist healthcare during ambulance transports. The topic of using pharmacological coercion outside mental hospitals in Norway seems to be sensitive and seldom discussed. However, given the need for physical coercion to ensure the safe transportation of patients who are not competent and display aggressive behavior, it is timely to initiate a discussion on patient welfare and the safety of all parties involved.

### List of abbreviations

NOT Nina Oeye Thorvaldsen, first author

OUS Oslo University Hospital

STC Systematic Text Condensation

TLH Tonje Lossius Husum, second author

RGP Regular General Practitioner

NAEMSP The National Organization of EMS Physicians

EMS Emergency Medical Services

USA United States of America

UK United Kingdom

### Consent for publication

Not applicable

### Availability of data and materials

The data that support the findings of this study are available on reasonable request from the corresponding author [NOT]. The data are not publicly available due to them containing information that could compromise research participant anonymity.

### Competing interests

The authors declare that they have no competing interests

### Funding

None

### Authors’ contributions

NOT conceived the study, designed the method, was the lead in the data acquisition and analysis process, and drafted and revised the manuscript. TLH contributed to the method, participated in the data acquisition and analysis, and revised the manuscript. SS contributed to the method, and the analysis, and revised the manuscript. All authors approved the final version of the manuscript.

### Author information

NOT was educated as a paramedic at OUS and has a bachelor’s degree in nursing. NOT has four years of work experience as a nurse and sixteen years of experience as a paramedic. The study was NOT`s master thesis in the study of Prehospital Critical Care (PHCC) at Stavanger University (UiS). NOT works as a PhD student in the Bachelor`s Degree Programme in Paramedic Science at Oslo Metropolitan University.

TLH is a Professor of Mental Health Care at Oslo Metropolitan University, Faculty of Health Sciences, and a clinical psychologist, and has worked on ethical questions relating to using coercion in mental health care services and carried out research on medical ethics at the Centre for Medical Ethics, University of Oslo. TLH has a PhD in the use of coercive measures in acute mental health departments.

SMJS is a trained physician specialized in anaesthesiology and has predominantly worked as a critical care physician in the Norwegian Helicopter Emergency Services since 2002. He has a PhD in patient safety and risk management and at the time of the study h was a Professor of Prehospital Critical Care at the University of Stavanger, and the chief medical officer of the Norwegian Air Ambulance Foundation.

##### Acknowledgements

In order to ensure independent reliability when selecting themes, an independent reviewer was asked to identify themes from the transcribed interviews. Those themes were then compared with the themes identified by the research teams. Many thanks to Johanne Jordahl (JJ) for her help with reading through the interviews.

## Appendix

1. Semi-structured interiewguide
2. The guidelines «Pasient som ikke transporteres til sykehus/lege»

### 1. Semi-structured Interviewguide

*Can you tell us about any experiences where you have used coercion prehospitally*?

*Can you tell us about other situations where you have use coercion?*

*How do you percieve coercion*

*How would you define coercion?*

*How is coercion performed by ambulance personnel?*

*Have you experienced unpleasant feelings or that it has been challenging to use coercion?*

*What have you experienced? Can you give examples?*

### 2. The guideline «Pasient som ikke transporteres til sykehus/lege»

**Pasient som ikke transporteres til sykehus/ lege**

**Pasient som ikke transporteres til sykehus/ lege**

**DEFINISJONER.**

De tilfeller der denne prosedyren vil komme til anvendelse, er:

1. Der ambulanse blir sendt ut og det viser seg at pasienten må̊

vurderes av lege for eventuell innleggelse i

sykehus. Det er ambulansepersonellet som må̊

sikre at pasienten faktisk kommer i kontakt med legen

2. De tilfeller der ambulansepersonellet vurderer at sykehusinnleggelse synes unodvendig og pasienten

våkner adekvat etter behandling, som ved

hypoglykemi hos kjent diabetiker

opiatoverdose hos kjent rusmisbruker

kramper hos kjent epileptiker uten medikamentell behandling

mindre uhell med svart moderat eller ingen pasientskade

astmatiker med god sykdomsinnsikt hvor symptomene forsvinner etter behandling, kliniske funn

stabiliseres og pasienten selv onsker å forbli på stedet

**Pasienten skal IKKE forlates alene**

**Ved tilstander ut over dette skal det konsulteres med lege dersom ambulansepersonellet ønsker å**

**forlate pasienten på stedet.**

3. De tilfeller der pasienten overlates til annet helsepersonell enn lege, for eksempel hjemmesykepleier

eller sykepleier på sykehjem eller annen behandlingsinstitusjon

4. I de tilfeller der pasienten overlates andre etater enn helsevesenet (f.eks. politi)

**BESKRIVELSE:**

Alle opplysninger vedrorende pasienten skal formidles på eget initiativ ved kontakt med legen

Når pasienten etterlates på stedet skal dette skje etter fullstendig klinisk undersokelse, eventuell

behandling og under forutsetning av at pasienten eller pårorende er informert om tilstanden, har forstått

den informasjonen som gis og samtykker

Med fullstendig klinisk undersokelse menes undersokelse/ tiltak i folge gjeldende anbefalinger på

behandlingssiden i MOM og ovrige prosedyrer i tjenesten

Fullstendig utfylt ambulansejournal skal overleveres pasient eller pårorende (dersom pasienten selv ikke

er beslutningskompetent) etter en komplett pasientundersokelse. Navn på personer det inngås avtaler

med (annet helsepersonell, andre nodetater, pårorende mv) og innholdet i avtalen skal dokumenteres i

journalen

Alle avtaler vedrorende videre oppfolging av pasienten skal skje over logget trafikkvei

Pasienten eller pårorende skal alltid informeres om å kontakte helsevesenet dersom pasientens tilstand

skulle endre seg, pasienten skulle få nye symptomer eller på annen måte fole seg uvel eller syk. Dette skal

dokumenteres i journalen

Pasienter som nylig har sokt helsehjelp flere ganger siste dager uten at tilstanden har bedret seg skal tas

med til lege dersom pasienten/ pårorende onsker det

Pasient som har eller har hatt brystsmerter skal ikke forlates på stedet uten legeundersokelse eller

legekonsultasjon selv om 12 kanaler EKG er negativ

## References

1. United Nations. Human Rights [Available from: https://www.un.org/en/about-us/universal-declaration-of-human-rights.

2. Grunnloven. Kongeriket Norges Grunnlov 1814 Accessed 14 Jun 2021. Available from: https://lovdata.no/lov/1814-05-17-nn.

3. NOU 2019: 14. Tvangsbegrensningsloven. Del 18- tvangsbegrepet.2019 14 Jun 2021. Available from: https://www.regjeringen.no/no/dokumenter/nou-2019-14/id2654803/.

4. Helsepersonelloven. Lov om helsepersonell m.v. LOV-1999-07-02-641999 Accessed 14 Jun 2021. Available from: https://lovdata.no/lov/1999-07-02-64.

5. Pasient- og brukerrettighetsloven. Lov om pasient- og brukerrettigheter. LOV-1999-05-21-302001 Accessed 14 jun 2021. Available from: https://lovdata.no/lov/1999-07-02-63.

6. Psykisk helsevernloven. Lov om etablering og gjennomføring av psykisk helsevern. LOV-2021-05-07-342021 14 Jun 2021. Available from: https://lovdata.no/lov/1999-07-02-62.

7. Helse- og omsorgstjenesteloven. Lov om kommunale helse- og omsorgstjenester m.m. LOV-2011-06-24-302011 Accessed 14 Jun 2021. Available from: https://lovdata.no/lov/2011-06-24-30.

8. Thorvaldsen NØ., Bergem AK., Holst Ø., Häikiö K. Coercion in the ambulance setting2022; 14. Available from: https://tidsskriftet.no/en/2022/10/short-report/coercion-ambulance-setting.

9. Dahlberg J. Arbeidet for å gi befolkningen et bedre helsetilbud er avhengig av et godt regelverk. Dette arbeidet kan ikke overlates til juristene alene.2022; 14.

10. § 7. Øyeblikkelig hjelp, LOV-1999-07-02-64 (1999).

11. Kjønstad A. Helserett, p. 153: Gyldendal forlag; 2007.

12. Kapittel 4 A. Helsehjelp til pasienter uten samtykkekompetanse som motsetter seg helsehjelpen mv., LOV-1999-07-02-63 (1999).

13. Psykisk helsevernloven. Lov om etablering og gjennomføring av psykisk helsevern, kap. 3. LOV-1999-07-02-621999 14 Jun 2021. Available from: https://lovdata.no/lov/1999-07-02-62.

14. Psyksisk helsevernloven. Lov om etablering og gjennomføring av psykisk helsevern, § 3-1. LOV-2021-05-07-34.2021 14 Jun 2021. Available from: https://lovdata.no/lov/1999-07-02-62.

15. Psyksisk helsevernloven. Lov om etablering og gjennomføring av psykisk helsevern, § 4-8. LOV-2021-05-07-34. 2021 14 June 2021. Available from: https://lovdata.no/lov/1999-07-02-62.

16. Straffeloven. Lov om straff, §§ 17-18. LOV-2022-04-08-22.2015 Accessed 14 Jun 2021. Available from: https://lovdata.no/lov/2005-05-20-28.

17. The Royal College of Emergency Medicine. Guidelines for the Management of Excited Delirium / Acute Behavioural Disturbance (ABD)2008. Available from: https://res.cloudinary.com/studio-republic/images/v1635412334/RCEM_Guidelines_for_Management_of_Acute_Behavioural_Disturbance_May2016/RCEM_Guidelines_for_Management_of_Acute_Behavioural_Disturbance_May2016.pdf?_i=AA.

18. Kupas D.F., Wydro G.C. Patient Restraint in Emergency Medical Services Systems. Prehospital Emergency Care [Internet]. 2009; 6(3):[340-5 pp.]. Available from: https://doi.org/10.1080/10903120290938436.

19. Burnett A.M., Panchal D., Peterson B., Ernest E., Griffith K., Frascone R.J., et al. The administration of pre-hospital ketamine for chemical restraint does not prolong on-scene times compared to haloperidol based sedation. The Australasian Journal of Paramedicine. 2015;12(1).

20. Tian L.L., Newman W.J. Psychiatric Considerations Regarding Prehospital Administration of Ketamine for Agitation. The Journal of Nervous and Mental Disease. 2019;207(1):43-4.

21. Isenberg D.L., Jacobs D. Prehospital Agitation and Sedation Trial (PhAST): A Randomized Control Trial of Intramuscular Haloperidol versus Intramuscular Midazolam for the Sedation of the Agitated or Violent Patient in the Prehospital Environment. Prehospital and Disaster Medicine [Internet]. 2015; 30(5):[491-5 pp.]. Available from: doi:10.1017/S1049023X15004999.

22. Cester-Martínez A., Cortés-Ramas J.A., Borraz-Clares D., Pellicer-Gayarre M. Inhaled Loxapine for the Treatment of Psychiatric Agitation in the Prehospital Setting: A Case Series. Clinical Practice and Cases in Emergency Medicine [Internet]. 2017; 1(4):[345-8 pp.]. Available from: DOI: 10.5811/cpcem.2017.5.33840.

23. Scaggs T.R., Glass D.M., Hutchcraft M.G., Weir W.B. Prehospital Ketamine is a Safe and Effective Treatment for Excited Delirium in a Community Hospital Based EMS System. Prehospital and Disaster Medicine [Internet]. 2016; 31(5). Available from: doi:10.1017/S1049023X16000662.

24. Ho J.D., Smith S.W., Nystrom P.C., Dawes D.M., Orozco B.S., Cole J.B., et al. Successful Management of Excited Delirium Syndrome with Prehospital Ketamine: Two Case Examples2012; 17(2):[274-9 pp.]. Available from: https://doi.org/10.3109/10903127.2012.729129.

25. Nilsen JE., Wik L., Kramer-Johansen J., Styrkson K., Tjelmeland IBM., Seland N., et al. Fremtidens prehospitale tjenester. 2014.

26. Spesialisthelsetjenesteloven. Lov om spesialisthelsetjenesten m.m. LOV-2022-06-10-35. 1999.

27. Brevig K.A. Sluttrapport transport av psyksisk ustabile personer- fase 1 (2015-2016). Sykehuset Innlandet HF; udatert.

28. Platou H-C.S., Hørthe K. Trygg og god transport av psykisk ustabile pasienter. Sluttrapport. 2016.

29. Raanes S.O. Prosjekt transporttilbud psykisk syke HMN RHF – Sluttrapport. In: RHF HM-N, editor. 2018.

30. Bremseth F. Psykiatriambulansen i Stavanger- Årsrapport 2017. In: Stavanger H, editor. 2018.

31. Leaux J. Årsrapport PA Bergen 2014-2018.xls. 2019.

32. Leaux J. Rapport PA - 2019.xls. 2019.

33. Malterud K. Systematic text condensation: A strategy for qualitative analysis. Scandinavian Journal of Public Health [Internet]. 2012; 40(8):[795-805 pp.]. Available from: https://journals.sagepub.com/doi/abs/10.1177/1403494812465030.

34. Malterud K. Qualitative research: standards, challanges, and guidelines. The Lancet [Internet]. 2001; 358:[483-8 pp.]. Available from: https://doi.org/10.1016/S0140-6736(01)05627-6.

35. Malterud K. Kvalitative forskningsmetoder for medisin og helsefag. 4 ed. Oslo: Universitetsforlaget; 2017.

36. Stalmejer R., McNaughton N., Van Mook W.N.K.A. Using focus groups in medical education research. Medical teacher [Internet]. 2014; 36(11):[1-17 pp.]. Available from: DOI:10.3109/0142159X.2014.917165.

37. Malterud K. Fokusgrupper som forskningsmetode for medisin og helsefag. Oslo: Universitetsforlaget; 2012.

38. Marti´n-Rodri´Guez L.S., Beaulieu M-D., Dámour D., Ferrada-Videla M. The determinants of successful collaboration: A review of theoretical and empirical studies. Journal of Interprofessional Care [Internet]. 2005:[ 132 – 47 pp.].

39. Gaisford M. Informed concent in paramedic practice. Journal of Paramedic Practice. 2017;9(2):80-5.

40. Roberts L, Henderson J. Paramedic perceptions of their role, education, training and working relationships when attending cases of mental illness. Journal of Emergency Primary Health Care. 2009;7(3).

41. Shaban R. Paramedic clinical judgment of mental illness: Representations of official accounts Journal of Psychosomatic Research [Internet]. 2005; 78:[529-35 pp.]. Available from: http://dx.doi.org/10.1016/j.jpsychores.2015.03.007.

42. Cutler D., Smith M., Wand T., Green T., Dinh M., Gribble R. Involuntary admissions under the Mental Health Act 2007 (New South Wales): a

comparison of patients detained by ambulance officers, medical practitioners and

accredited persons in an emergency department. Emergency medicine Australasia : EMA [Internet]. 2013; 6:[254-9 pp.]. Available from: http://dx.doi.org/10.1111/1742-6723.12138

43. Rees N., Rapport F., Snooks H., John A., Patel C. How do emergency ambulance paramedics view the care they provide to people who self harm?: Ways and means. International Journal of Law and Psychiatry [Internet]. 2017; 50:[61-7 pp.]. Available from: http://dx.doi.org/10.1016/j.ijlp.2016.05.010.

44. Pelto-Piri V., Kjellin L., Hylén U., Valenti E., Priebe S. Different forms of informal coercion in psychiatry: a qualitative study. BMC Research Notes [Internet]. 2019 2019/12/02; 12(1):[787 p.]. Available from: https://doi.org/10.1186/s13104-019-4823-x.

45. National Highway Traffic Safety Administration. Fourth report to Congress: effectiveness of occupant protection systems and their use. Washington, DC: US Department of Transportation, National Highway Traffic Safety Administration. 1999.

46. Høye A., Elvik R. Bilbelter, beltepåminnere og beltelås i lette kjøretøy: Transportøkonomisk institutt. Stiftelsen Norsk senter for samferdselsforskning. ; 2015 [Available from: https://www.tshandbok.no/del-2/4-kjoeretoeyteknikk-og-personlig-verneutstyr/doc684/.

47. Steiro A., Dahm KT., Hilde Strømme H., Brurberg KG. Tvangsmedisinering i psykisk helsevern – en systematisk kartleggingsoversikt2018. Available from: https://www.fhi.no/publ/2018/tvangsmedisinering-i-psykisk-helsevern--en-systematisk-kartleggingsoversikt/.

48. Ness E., Skotte J.R., Christensen T.B., Andresen J.F. Kan vi redde flere liv?2020. Available from: https://tidsskriftet.no/2020/05/kronikk/kan-vi-redde-flere-liv.

49. Haaland V.Ø., Bjørkholt M., Freuchen A., Ness E., Walby F.A. Selvmord, psykisk helsevern og tverrfaglig spesialisert rusbehandling i Agder 2004 –13. Tidsskr Nor Legeforen. 2017.

50. Sykehuset i Vestfold HF og Sykehuset i Telemark HF. Prosjekt Verdig vei videre- transport av psyksisk syke i akutt krise.

51. Helse Sør-Øst. Regional sluttrapport 2017. Prosjekt «Transport av psykisk ustabile personer». 2017.

52. NOU 2019: 14. Tvangsbegrensningsloven. Del 11- Relevant nasjonal og internasjonal kunnskap om forebygging av tvang 2019 [Available from: https://www.regjeringen.no/no/dokumenter/nou-2019-14/id2654803/?q=redusere&ch=3#kap11.

53. Helsedirektoratet. Nasjonale faglige råd for forebygging og riktig bruk av tvang i psykisk helsevern for voksne. Oslo: Helsedirektoratet; 2021 [updated 25 Marz 2021, accessed 16 May 2021. Available from: https://www.helsedirektoratet.no/faglige-rad/tvang-i-psykisk-helsevern-for-voksne-forebygging-og-riktig-bruk.

54. Shaban R.Z. Mental health and mental illness in paramedic practice: A warrant for research and inquiry into accounts of paramedic clinical judgment and decision-making. Journal of Emergency Primary Health Care (JEPHC) [Internet]. 2004 9 Jul 2022; 2(3-4). Available from: https://ajp.paramedics.org/index.php/ajp/article/view/285/286.

55. May S. The ethical and legal dilemmas paramedics face when managing a mental health patient. Journal of Paramedic Practice 2016;9(1).

56. Townsend R., Luck M. Protective jurisdiction, patient autonomy and paramedics: the challenges of applying the NSW Mental Health Act. Journal of Emergency Primary Health Care (JEPHC). 2009;7(4).
